# Internet-of-Things and Information Fusion: Trust Perspective Survey

**DOI:** 10.3390/s19081929

**Published:** 2019-04-24

**Authors:** Farag Azzedin, Mustafa Ghaleb

**Affiliations:** Information and Computer Science Department, King Fahd University of Petroleum and Minerals, Dhahran 31261, Saudi Arabia; g200905270@kfupm.edu.sa

**Keywords:** information fusion, trust, reputation, Internet-of-Things, wireless sensor networks

## Abstract

The advent of Internet-of-Things (IoT) is creating an ecosystem of smart applications and services enabled by a multitude of sensors. The real value of these IoT smart applications comes from analyzing the information provided by these sensors. Information fusion improves information completeness/quality and, hence, enhances estimation about the state of things. Lack of trust and therefore, malicious activities renders the information fusion process and hence, IoT smart applications unreliable. Behavior-related issues associated with the data sources, such as trustworthiness, honesty, and accuracy, must be addressed before fully utilizing these smart applications. In this article, we argue that behavior trust modeling is indispensable to the success of information fusion and, hence, to smart applications. Unfortunately, the area is still in its infancy and needs further research to enhance information fusion. The aim of this article is to raise the awareness and the need of behavior trust modelling and its effect on information fusion. Moreover, this survey describes IoT architectures for modelling trust as well as classification of current IoT trust models. Finally, we discuss future directions towards trustworthy reliable fusion techniques.

## 1. Introduction

Internet-of-Things (IoT) is emerging as a vital tool for innovation where data sources, composed of tiny internet-connected devices, generate data and, hence, enable smart applications [1,2,3]. These devices disseminate data which is collected and utilized by smart applications. The real value of these IoT smart applications comes from analyzing the information provided by these devices. Fusing information across multiple devices can enable or enhance information completeness and hence, service quality. The collected sensor-generated datasets are prone to factors that render the datasets unreliable. These factors can be related to technical or behavior issues. Technical issues, such as the deployment environment conditions and transmission impairment, are investigated and addressed by many researchers [4,5,6]. These technical issues focus on details such as verifying the authenticity of a device and determining the authorizations that the device is entitled to access. Used techniques include encryption, data hiding, digital signatures, authentication protocols, and access control methods.

On the other hand, behavior issues for IoT devices have just recently started to get attention from the research community [1,7,8,9,10,11,12]. Having created a smart environment, the IoT vision is to allow different sensors to disseminate vital data including the human body and environmental measurements. These measurements do not only monitor their environments, but also perform effectively in shared tasks and influence decision-making processes in other environments. We cannot assume that all devices are trustworthy, honest, and accurate. If these sensors act maliciously, catastrophes may occur in these already complex IoT systems. Furthermore, and since these devices closely affect human life, disastrous consequences are posed by injecting false data [8,12].

Handling behavior-related issues are complicated due to site autonomy, data source heterogeneity, distributed ownership, and diverse resource clients [13]. Behavior trust deals with a wider notion of a device’s *trustworthiness*. A malicious tiny device could deliver the wrong data or even refuse to participate. A digitally signed certificate is incapable of conveying if the sender is malicious and a digitally signed code does not ensure if the code is written by a trustworthy programmer.

Therefore, building trusted IoT environments is of great importance to achieve the full benefits of smart applications. In addition, building trusted IoT environments mitigates unrecoverable and unexpected damages in order to create reliable, efficient, stable and flexible smart sensor-driven systems. Hence, ensuring trust will provide the confidence and belief that IoT devices and consequently IoT services perform as expected.

Information fusion phases can be applied on sensors-generated information to ensure data reliability [8,14,15,16]. Information fusion techniques can combine data from multiple data sources to increase data reliability. Unfortunately, a data source can be dishonest and therefore generates a bogus data. Even if the data source is honest, the data source can be inaccurate and therefore received data needs to be adjusted. Furthermore, a data source can be untrustworthy. That is, we want to ensure that our sink device, where the data analysis and fusion processes are done, is trustworthy (i.e., does not modify the collected datasets nor the fused data).

This motivated us to (a) clearly outline that trust management systems should be an integral part of any information fusion process. That is, emphasising that malicious behaviour has a negative effect on information fusion processes and thus on emerging smart applications, (b) conduct an extensive survey on IoT architectures for trust modelling, (c) conduct an extensive survey on IoT trust models, and (d) propose a novel trust model taxonomy. Based on our extensive survey, the research community needs to explore this vital factor that renders data sources and hence, collected datasets unreliable. In fact, when a malicious device *x* is providing wrong data and node *y* is interested in this data, then *y* will allocate some of its resources to accept and process the wrong data. These wasted allocated resources as well as *y*’s processing time are all indirectly consumed and eventually wasted by *x* in an environment where we need to limit energy consumption for these tiny smart devices. In addition, *x*’s malicious behavior negates the advantages of distribution and sharing nature. Device *x* also contributes negatively to the active role of contributing devices as they become overloaded and this might encourage selfishness. A malicious contributor fits the classification of free riders [17] because such a device discourages trustworthy contribution. In a nutshell, a malicious device does not only fit the classification of a free rider, but also introduces new challenges to the success of smart IoT applications.

In this article, we argue that only trustworthy and honest data sources should be utilized in order to achieve the full benefits of smart IoT applications. That is, before intermediate devices start the data fusion process, data sources as well as data itself need to be filtered to achieve the full benefits of smart applications.

For completeness purposes, Section 2 discusses the fundamental concepts that need to be considered when modeling trust. Section 3 elaborates the need for trust-based information fusion while Section 4 sets the stage with the background information. Section 5 reviews some recent studies in information fusion field that address the issue of trust. The current IoT architectures for modeling trust is presented in Section 6. Section 7 classifies the existing IoT trust models according to trust design parameters while Section 8 outlines IoT trust models. Finally, Section 9 and Section 10 concludes the article and envisions future directions, respectively.

## 2. Fundamental Trust Model Concepts

Trust models [12,13,18,19,20] are proposed to tackle behaviour-related issues. Recently, behavior issues have started to get attention from the information fusion research community [21].

Trust is conceptualized in diverse ways and there are many trust models discussed in the literature [18,19,22,23,24,25,26,27]. These trust concepts are not our focus in this article since we are not proposing a trust model but rather: (a) argue and raise the awareness for trust-based information fusion processes and (b) outline malicious behavior’s effect on information fusion processes and thus on emerging smart IoT applications. Thus, the trust concepts discussed in this section are not comprehensive and we include them only for completeness purposes.

### 2.1. Behavior Trust

Behavior trust is identified as a vital component in any Internet-based transaction and lack of behavior trust is a major obstacle for the potential growth of Internet communities [12,13,18,19,20]. Operating in open and dynamic environments, a device encounters unfamiliar and possibly hostile devices. There is a lack of consensus in the literature on the definition of behavior trust and on what constitutes behavior trust management [12,13,18,19,20]. Trust is a multi-dimensional notion that is suitable for a wide range of relationships [12,13,18,19,20]. Researchers have defined trust in different ways, which often reflects the researcher’s background. The definition of behavior trust used in this article is adopted from [18,19]:

A device is trustworthy if there is a firm belief in the competence of the device to act as expected such that this firm belief is not a fixed value associated with the device but rather it is subject to the device’s behavior and applies only within a specific context at a given time.

### 2.2. Reputation

In a dynamic setting, devices need to manage risks involved in interacting with other devices. In a dynamic environment, devices are vulnerable to risks because of unknown, incomplete, or distorted information about each other. One way to address this problem is to establish trust through reputation. The reputation concept is already used in trust models. When making trust-based decisions, devices can rely on others for information pertaining to a specific device. For example, if device *x* wants to make a decision about whether to engage in a transaction with device *y*, which is unknown to *x*, *x* can rely on the reputation of *y*. The definition of reputation used in this article is adopted from [18,19]:

The reputation of an entity is an expectation of its behavior based on other entities’ observations or the collective information about the entity’s past behavior within a specific context at a given time.

Forming the reputation of a device so that it is effective, informed, and reflects the device’s trustworthiness depends on two factors: (a) the honesty of the information source (i.e., the device that sent the information) and (b) the accuracy of the information received. Therefore, the objective is to rely on devices that are honest as well as to rely on information that is accurate. Honesty and accuracy are defined next.

### 2.3. Honesty

Relying on other devices for information when seeking the reputation of device *y*, device *x* might be misinformed and form the wrong perception about *y*. This is due to dishonest recommenders that try to pollute the environment by intentionally giving bogus reputation reports. Ideally, we want to prevent dishonest devices from contributing to the computation of reputation. The definition of honesty used in this article is adopted from [18,19]:

A recommender is said to be honest if the information, pertaining to a specific entity within a specific context at a given time, received from entity is the same information that entity believes in.

When device *x* gives out information, *x* fetches this information from a data structure that *x* maintains. Hence, by believes in, we mean the information that is stored in *x*’s data structure. Honesty is a critical factor in any trust model. In modeling trust, we can not assume that devices are honest and therefore we need a mechanism to identify and prevent dishonest devices from polluting the recommendation network. Hence, the goal is to come up with a mechanism to compute honesty and use this measure to weed out and prevent dishonest recommenders from influencing the recommendation network.

### 2.4. Accuracy

The objective here is to ensure that the received information pertaining to device *y* is as close as possible to the trustworthiness of *y*. The definition of accuracy used in this article is adopted from [18,19]:

A recommender is said to be accurate, if the deviation between the information received from it pertaining to the trustworthiness of a given entity y in a specific context at a given time and the actual trustworthiness of y within the same context and time is within a precision threshold.

A trust model uses the accuracy notion as a measure that is applied to the information received from a recommender to infer what the recommender really means. This scaling process takes into account how accurate the recommender’s advertisement is. It should be noted that accuracy is an independent notion from a recommender’s trustworthiness.

## 3. The Need for Trust-Based Information Fusion

Information fusion is a process that allows us to estimate and assess situations by collecting diverse and sometimes conflicting information from various information sources. This process involves integrating information from these multiple information sources to produce specific and comprehensive yet unified estimate about the situation. Multiple information sources such as sensors generate information which is collected by sink node(s), where the information fusion processes take place. These processes may include transformation, reduction, integration, or replacement [8,14,15,16]. Finally, an output is generated that is hopefully more useful than that provided by any individual information source.

Key challenges during this information fusion process arise from dealing with information sources’ behaviour issues such as trustworthiness, honesty, and accuracy. These challenges are many folds. First, an information source or a sink device can be untrustworthy. That is, they can intentionally modify the collected datasets or fused information. Second, a dishonest information source can intentionally lie for their own benefit and thus submit wrong information. Third, honest information sources have different levels of accuracy relating to their imprecise individual trust metrics. These different levels of accuracy arise because nodes can evaluate the same situation differently. As such, the concept of accuracy enables review-based mechanisms to function with imprecise metrics.

Therefore, before relying on the fused information, we must ensure trustworthiness, honesty, and accuracy for our information sources and also for our sink devices. In a nutshell, we want to avoid the problem of fusing untrustworthy or dishonest information which we believe is a key factor to achieve the full benefits of smart applications.

## 4. Background

### 4.1. Information Fusion, IoT, and Sensors

Information fusion is composed of two steps, namely pre-classification and post-classification fusion. Pre-classification fusion deals with combining the information prior to the application of any classifier or matching fusion algorithm [28]. During the post-classification fusion phase, the information is combined after the decisions of the classifiers have been obtained [28].

The emergence of IoT vision comes as a result of the integration of different enabler technologies together such as sensory, communication, service-oriented architecture, networking, and intelligent information processing technologies. IoT systems have a wide range of implications and objectives. One of these objectives is to interconnect different objects such that these objects are addressable, locatable, and readable. Furthermore, each IoT object can be used either to fulfill specific goals or produce and consume IoT services [29]. These IoT objects vary from limited sensing capability sensors such as wearable devices, consumer goods, complicated endpoints, and advanced systems [30].

According to the 2020 conceptual framework [31], IoT architecture needs four components as expressed in Equation (1):

(1) IoT=Services+Data+Networks+Sensors

**Figure 1:**
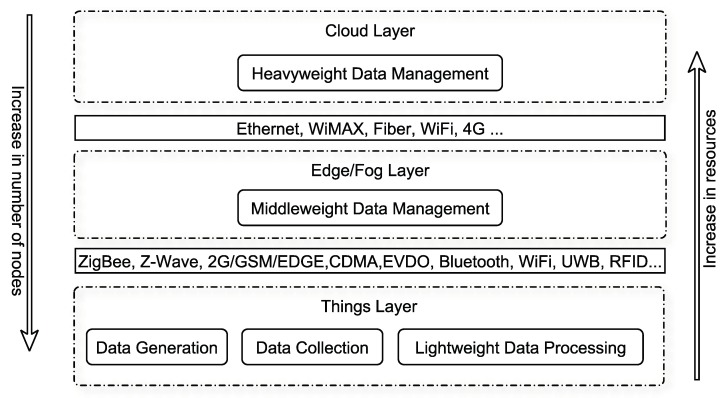
A General architecture of Internet-of-Things.

Sensors, as data sources, form Wireless Sensor Networks (WSNs) and are an integral part of IoT environments. Sensors are located at the bottom layer (i.e., the things layer), where data is produced and consumed as shown in Figure 1. An edge layer, also known as fog layer, uses edge devices to perform a substantial amount of computation, storage, and communication to ensure seamless accessibility, reliability, and timeliness of the data for its users. Powerful devices are located at the Cloud layer to perform heavyweight tasks such as data mining. Services are provided to users through IoT applications in a ubiquitous manner [32].

Reliability of smart applications is threatened due to the lack of standard methods for addressing their data sources trust concerns [33]. To achieve the full benefits of smart applications, we have to manage the trust of data sources. For example, data sources can act deceitfully by providing misleading or false information and this type of threat cannot be mitigated using traditional security mechanisms [13]. Trust, unlike security, is concerned with sensors’ features or attributes such as reputation, honesty, and accuracy [17,34].

As most IoT sensors have been used to either monitor or perform some actions to a large number of time-critical decision-making systems, any malicious behaviour of such sensors may result in harmful consequences. For example, the malicious behaviour of smart vehicles could put passengers’ safety in danger. Therefore, trustworthiness is an indispensable requirement to mitigate such risks in such systems [13,35]. A successful trust management system detects malicious activities, improves the success rate of information fusion techniques, and hence, provides qualified and reliable smart applications.

### 4.2. Possible Trust Threats Affecting Smart Applications

IoT environments basically incorporate a considerable amount of heterogeneous sensors to provide various broad smart applications. Some of these sensors can be untrustworthy and carry out trust-related attacks for the sake of their self-interest while others can boost their allies to collaboratively attack a particular service provider in order to ruin its reputation and increase the reputation of each other.

In the context of information fusion, our concern is trust-related threats which render smart applications unreliable. Smart applications rely heavily on generated data and fused data. Data is generated by data sources (i.e., sensors) and transmitted to data sinks, where the data fusion process takes place [13,36,37,38]. Trust systems should model and maintain trust level for these various data sources as well as data sinks to mitigate possible trust-related attacks. Ballot-stuffing, bad-mouthing, self-promoting, and opportunistic service attacks are considered as the most popular types of trust-related attacks [8,13,20]. Other types of attacks include collusion, on-off, whitewashing, and discriminatory attacks. These attacks are utilized by untrustworthy or dishonest sensors to evade detection. In the literature [13,20,39], these attacks are defined in Table 1.

### 4.3. Major Challenges of Trust Models

Heterogeneity is considered a significant challenge to sensor-based system design for two reasons. First, computational power and storage capacity vary from IoT device to another such as RFID tags, sensors, laptops and smartphones. Second, connectivity method to the network varies from device to device such as Wi-Fi, cables, 3G, Bluetooth, and near field communication. Trust models manage the trust for these tiny wireless sensors located at the things layer of IoT systems. These devices have limited computation, storage, and power resources. This is a big challenge for IoT trust models. Also, sensor-based devices with various capabilities from various manufacturers must be able to communicate. As a consequence, existing trust protocols do not perform well to adapt this requirement [9,13,38].

The dynamicity nature of sensor-based systems caused by the continues joining and leaving of sensors as well as smart applications is another challenge that needs to be considered when modeling trust [40]. In addition, sensor mobility can also create several challenges in terms of network and protocol efficiency [41].

The growing nature of sensors should be considered when designing trust models. As stated in [42,43], there are 9 billion interconnected sensors and this number is expected to reach 20 billion by 2020. Hence, a trust model for smart applications should be scalable [7,44].

The extensive daily use of smart applications by humans is another challenge. Trust models have to consider social relationships among device holders and QoS attributes [13,38] to increase the success of these sensor-based smart applications.

## 5. Trust-Based Information Fusion

Information is vital in enhancing smart IoT applications. Sensors observe their environment by gathering and disseminating data to decision making nodes [45,46,47,48]. However, some information sources might be malicious when they share their opinions. Along these lines, decision making nodes should wipe out opinions gathered from such malicious information sources [48,49].

Hence, many trust-based fusion methods are suggested in the literature. Some methods use only the subjective logic’s cumulative fusion operator [50,51] to fuse trust opinions. These methods do not consider the trustworthiness of information providers before fusion. For example, no discount of opinions is performed before fusion. Other methods estimate the trust of information sources using threshold [51] and then discount and fuse the opinions. Subjective logic discounting and cumulative fusion operators are utilized in order to form the fused opinion.

During information fusion, some approaches model the trustworthiness of information providers and utilize the estimated trust in order to discount opinions. For example, Jøsang and Ismail [52] introduced a reputation system to assess the likelihood of a proposition utilizing probability density functions. As input parameters to their system, they considered a beta distribution with collected ratings of information sources. An extension to [52] is done by Whitby et al. [53] to manipulate misleading opinions from malicious information sources using a majority-based filtering technique. This method expects that malicious sources are in minority and hence, are ineffective. To overcome this limitation, another method proposed by Teacy et al. [54] utilizes individual observations about information sources to estimate their trustworthiness. However, lacking evidence about historical behaviour of information sources will make this method ineffective [48].

Other fusion methods in the literature consider various behaviors of malicious information sources and exploit them during fusion. These methods consider just the expected behaviors’ probabilities during fusion. For example, the Hierarchical And Bayesian Inferred Trust model (HABIT) [55] and Bayesian Learning to Adapt to Deception in E-Marketplaces (BLADE) [56] exploit the change of information sources’ behavior (such as flipping their opinions). These methods empower a node to utilize all available reports with minimizing the need for discounting or discarding opinions even if the opinions sources are viewed as malicious.

Venanzi et al. [49] proposed a trust-based fusion technique to estimate recommenders’ honesty. This approach integrates trust into the fusion process to estimate the honesty of its recommenders. A likelihood model of the trustworthiness of nodes is used to scale the uncertainty of multiple estimates with parameters of trustworthiness. Etuk et al. [21] proposed a trust-based information fusion approach by diversifying the set of information sources to increase the heterogeneity of information providers and hence, reduce collusion attacks.

Authors in [46] utilize trust-based information fusion strategy to appraise the trustworthiness of the information sources. This strategy utilizes subjective logic for discounting and fusing the source opinions. The strategy uses a nonlinear constrained optimization algorithm to detect and resolve conflicts during the discounting process.

Kaplan et al. [51] proposed an approach to derive trust evidence of information providers by exploiting conflicts and consistencies between individual observations of information providers. This approach starts by using the derived trust evidence to model the trustworthiness of information providers. The trustworthiness of the information providers is used to filter unreliable observations. During the aggregation process, a subjective logic cumulative fusion operator is used to fuse the filtered observations.

Table 2 classifies trust-based information fusion based on different dimensions. Information fusion techniques can utilize trust models either in the selection [21,45,46,51,57,58,59] or in the information fusion phase [49,52,53,60]. That is, the decision maker can select the information providers based on trust. On the other hand, the decision maker can integrate trust during the fusion process. Another classification dimension is to identify the target node for which trust is computed. Some information fusion techniques compute trust of the information sources because their objective is to filter recommenders [21,45,46,49,51,52,53,57,58,59,60]. Other information fusion techniques compute trust of the aggregator node(s) [60]. Once reports are collected by the decision maker, these reports can be customized by a decaying or discounting function [21,45,46,51,60] before fusing them using various fusion operators.

## 6. Internet-of-Things Architectures for Modeling Trust

### 6.1. Centralized Architectures

Authors in [33] proposed a centralized architecture for trust evaluation in IoT. They utilized the publish-subscribe paradigm in which service providers (publishers) publish the sensor data to the broker and service consumers (subscribers) receive notifications related to their interests from the broker. In their approach, trust computing and prediction module is placed on the cloud. Authors in [61] proposed a centralized architecture for modelling their proposed trust model. In this architecture, IoT objects must register with a centralized service server and publish their services. A centralized trust management server receives feedback from these objects after every transaction. This server computes and stores the reputation and contextual trust values. The same authors enhanced their proposed work in [33] by using a clustered architecture [62] to reinforce security as well as minimize the number of stored trust values on each constrained object. It should be noted that [62] still utilized centralized servers to manage the trust.

A centralized architecture was proposed in [63] where a centralized trust entity maintains trust levels of IoT objects and chooses the best capable object to serve a request. Authors in [64] proposed a centralized architecture for modeling trust where a central server calculates, stores, and updates trust values of the IoT objects. Service and path discovery are also achieved through a centralized database server.

In [65], authors proposed a centralized architecture for trust management of SIoT that incorporates recommendation, reputation and knowledge trust metrics. Recommendation metric denotes the opinions of trustor-related entities to the trustee. Reputation metric is utilized to maintain the global opinions on the trustee. The knowledge metric represents the provided information by the trustee to assess its trustworthiness based on trust metrics such as cooperativeness, honesty, experience, and community of interest. To deal with the scalability issue, they have suggested to utilize fog-based architecture. However, details of the architecture are not covered and many trust and social relationships metrics are overlooked in this study.

An architecture to manage trust is proposed in [66]. The proposed system consists of distributed trust agents for producing and filtering trust parameters. These trust parameters are managed in a centralized manner. In [67], authors proposed a centralized trust management architecture that contains a supernode to serve as a centralized trust manager. The IoT system is divided into clusters, where the supernode stores the trust values of all IoT objects in a central repository.

IoT trust (IoTrust) architecture is proposed in [20]. This architecture integrates *software defined network* for IoT and proposes five layers namely reputation management layer, organization layer, *software defined network* control layer, node layer, and object layer. The node and organization reputations are evaluated at a reputation management centralized repository.

### 6.2. Distributed Architectures

In [68], authors introduced a distributed IoT architecture for modeling trust. In this architecture, trust management is performed on every IoT object. In [69], the same authors improved their architecture presented in [68] by making each object stores and updates only trust values of other objects of interest in order to minimize the computation and the storage cost.

A distributed architecture [70] for modelling trust is proposed to utilize a decentralized approach that consists of objects providing services and feedback trust values. A decentralized bulletin board holds encrypted feedback and the reported non-interactive zero-knowledge proof by interacting machines. The reputation scores for machines are computed in a distributed manner and feedback values reported to the decentralized public bulletin board.

Authors in [7] proposed their blockchain-based architecture for modeling trust to enable IoT nodes to store tamper-proof trust information. In this paradigm, each node stores the full data of the entire system creating unnecessary redundancy. Furthermore, trust values must be validated by miners to ensure a consensus of data. Also, blockchain offers a tamper-proof data structure and not an efficient structure with respect to storage footprint and lookup time [71,72,73,74].

### 6.3. Hybrid Architectures

The hybrid scheme is a mix of centralized and distributed approaches to overcome the limitations of these approaches. A 3-tier (cloud-cloudlet-device) architecture is proposed in [44] to propagate trust values to a central cloud. This architecture facilitates the report/query of trustworthiness of IoT devices from the local cloudlets. Although this approach used a hybrid method, it uses a central cloud for propagating the trust values assembled from one cloudlet to the other cloudlets.

### 6.4. Summary

Table 3 summarizes the existing architectures for modeling trust in IoT environments. Trust information records are stored either in the things, the fog, or the cloud. In the case of using an ordinary thing to store this information, critical constraints are applied when the candidate thing is selected. These constraints relate to power and computation capabilities, thing availability, and link quality metrics. As shown in Table 3, two architectures are suggested when an ordinary thing is used as a directory; centralized and distributed. With regards to the use of fog node as a directory, three architectures are suggested, namely centralized, distributed, or hybrid. Fully centralized architecture is suggested when the cloud is utilized for storing the trust information.

From Table 3, it can be noticed that the centralized and distributed trust architectures have been considered thoroughly, while the hybrid architecture received insufficient consideration [13]. To the best of our knowledge, no work has been done for taking the advantages of centralized and distributed architectures except the study proposed by [44] which utilizes a cloudlet as an intermediate layer for storing trust information.

## 7. Taxonomy of Trust Models

**Figure 2:**
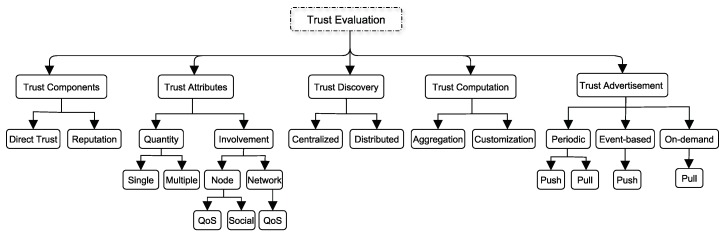
Taxonomy of Internet-of-Things Trust Models.

Based on our extensive survey of IoT trust models, we classify trust models into the taxonomy represented in Figure 2. This taxonomy classifies trust models based on five dimensions. The first dimension in our taxonomy is what are the trust components used to evaluate trust. A node can rely on its own experience or on recommendations to evaluate the trust levels of other nodes. The second dimension is to know which trust attributes are used and whether these attributes are associated with the node itself or the network. The third and fourth dimensions are concerned with how trust is discovered and computed in the trust model. Trust discovery can be centralized or distributed whereas different aggregation and customization methods can be used to compute the trust level. The final dimension answers the question of when trust levels are disseminated or advertised.

Before sensor *x* engages in any behavior trust relationships with target sensor *y*, *x* needs to evaluate the trust level of *y* which we will refer to as $TL_{y}$. Sensor *x* can rely on its own experience with *y* or the reputation of *y*. Therefore, trust has two components namely, direct trust and reputation. It should be noted that *x* can use both of these components to form $TL_{y}$.

Sensor *x* needs also to determine the quantity of trust attributes (either a single attribute or multiple attributes) as well as whether these are network or node attributes. For example, *x* can use two attributes such as honesty and packet delivery ratio to determine $TL_{y}$. In this case, honesty pertains to node *y* itself but packet delivery ratio pertains to the network. Node attributes can be QoS or social attributes whereas network attributes are only QoS-related attributes.

Trust discovery is concerned with trust revelation schemes. In general, the nodes that store and reveal trust levels are either distributed or centralized. A centralized trust discovery scheme requires a centralized node that can be represented by either a physical or a virtual node. This centralized node stores and/or reveals trust levels. Distributed trust discovery refers to having multiple nodes storing and/or revealing trust levels. This means that (a) trust levels are stored either on a centralized or distributed node(s) and (b) a centralized or distributed node(s) can reveal trust levels. In a nutshell, trust discovery is concerned with the nodes that store and reveal trust levels.

On the other hand, trust advertisement is concerned with when should a node reveal trust levels. Trust advertisement is initiated by a node to propagate trust levels to other nodes. In a periodic approach, trust levels are periodically disseminated from the originating node to other node(s) in which case it is pushing the trust levels. On the other hand, other nodes can request the trust levels in which case they are pulling the trust levels from the originating node. Trust levels can also be advertised based on events. For example, after every transaction, the trust level is pushed from the originating node to other nodes. In an on-demand approach, trust levels are advertised from the originating node immediately once it is demanded (requested) by other nodes. In this case, the information is pulled by the other nodes.

Trust models can be classified also based on the trust computation phase. That is, how the target sensor’s final trust level is computed. Once all the trust levels are collected, they need to be combined. For example, *x* can request its recommends for their trust levels about *y*. Then, *x* needs to compute $TL_{y}$ by combining all the trust levels collected from the recommenders. This combination process involves the usage of aggregation methods such as fuzzy logic, static/dynamic weighted sum, Bayesian inference, or regression analysis.

Once $TL_{y}$ has been formed, *x* can apply a measure to infer what $TL_{y}$ really means. This scaling process can apply a decay function to adjust $TL_{y}$. For example, if *x* has not interacted with *y* for sometime, *x* may decide to decay $TL_{y}$ to reflect the elapsed time factor since the last interaction. In the same manner, if the trust levels received from the recommenders are based on old transactions, *x* might decay these trust levels to infer what the recommender really means. So, in this case, *x* will adjust recommendations based on the accuracy of the recommender’s advertisement.

## 8. Internet-of-Things Trust Models

An IoT application can be represented as a mix of social networks, P2P MANETs, and service computing systems. These IoT devices dependently establish social relationships based on the nodes’ QoS as well as owners’ social network. Hence, trusted IoT devices are sought to offer needed services in both the cyberspace and physical world [13,39]. So, to build a trust model for IoT, QoS and social attributes of IoT devices and their owners have to be considered to establish an effective IoT trust model [13,34,39]. As such, this section is organized in subsections that cluster IoT trust models according to used trust attributes.

### 8.1. QoS-Social-Driven Trust Models

According to Refs. [68,76,77] trust assessment, evaluation and management depend on interactions among objects in social networks resulting in a distributed, encounter-based computations. Trust in such models is extracted based on social perceptions such as recommendation, reputation, and involvement by spreading knowledge between objects. The key disadvantage of these reputation methods is the need of human involvement for inputs. Together with the online transactions, the reputation methods can be used in MANETs, WSNs, and P2P systems that utilize interactions among objects that are spread over a network. For instance, various trust-based routing protocols in MANETS and WSNs evaluate the trustworthiness of a machine in the network by taking into account third-party thoughts and reputation besides their own knowledge to assure that a machine or a node will not get compromised.

In the literature, SIoT metrics have been used in trust computation phase to effectively manage trust in IoT environments. In [68], authors proposed a first distributed trust management model based on social relationships between owners of IoT devices. They consider three trust metrics: honesty based on node’s experiences, cooperativeness that is identified by the number of common friends, and community-based on the degree of common interest or similar capabilities.

Atzori et al. [78] presented a new approach for social network intelligent objects derived from SIoT based on subjective trust management model. Analogous to social networks for people, they defined a social network of intelligent objects that are linked with social relationships between objects. Their research work was inspired by trust in P2P networks. The bottom line of trust calculation depends mainly on a node’s knowledge and reputation among its known friends. Additionally, they developed a feedback system in which they combine the importance of the participated nodes and the trustworthiness. Furthermore, authors in [77] proposed a subjective trustworthiness evaluation model in SIoT environments. The computation of trust is performed at each node based on direct trust such as its own experiences and indirect trust such as the recommendation of other common friends towards a service provider. In the trust evaluation, they consider centrality as a measure of node willingness, node capabilities as a measure of context information, transaction relevance and other social relationships. Nitti et al. [79] proposed a hybrid trust model for SIoT applications. Both QoS and social trust attributes are considered in this model. QoS trust attributes include computational capability and transaction service quality, while social trust attributes include credibility, centrality, and relationship factors. In this model, they consider features from both distributed subjective and centralized objective trust models. In the distributed model, each node computes its own subjective trustworthiness towards another node based on its own experience and on the recommendations of its friends. In the centralized model, they proposed to propagate the trust assessments to centralized pre-trusted objects that maintain a distributed hash table to answer trust related inquires for a service provider. They assumed that pre-trusted objects are dedicated to answering inquiries and will not offer any services themselves.

Another adaptive distributed trust model is proposed by Rafey et al. [80] to enhance cooperation between trusted nodes and adjust the trust scores dynamically based on the node behavior. In this model, node transaction attributes (e.g., node computation power, confidence, context importance, and feedback), and node social attributes(e.g., friendship, centrality, and relationship) are considered. In the trust computation phase, each node computes the overall trust values of other nodes based on its own direct interactions and recommendations from other nodes. Also, their model integrates the social relationships and context of interactions in the trust computation. The trust accuracy in this model can be affected by recommendations from dishonest nodes that assign higher trust values to their group of allies.

Bao and Chen [76] proposed an event-driven trust model for IoT and they consider QoS and social trust attributes for trust computation. However, a context-aware issue has not been considered in their model. An access service recommendation model is proposed by Chen et al. [81] for effective service composition and resistance against trust-related attacks. In their model, they consider both QoS trust metrics including energy status and quality reputation and social trust metrics based on social similarities. However, this study doesn’t consider the contextual and dynamic nature of trust. Another study for trust management in SOA-based IoT application to service composition is proposed by Chen et al. [82]. In their model, they utilized service quality as a QoS attribute to rate a service provider and social attributes to rate a recommender. Aggregating self-observations is done by utilizing Bayesian inference, while aggregating recommendations is done by utilizing social similarity weighted sum. Although the authors used QoS to rate service provider and social attributes to rate recommenders, only service quality attribute is used in trust formation.

Khani et al. [11] proposed a contextual SIoT trust model including independent and dependent metrics. Independent metrics include advertised and expected QoS while dependent metrics include social similarity friendship, social similarity relations, social similarity community, and contextual feedback of trust. Also, they proposed a mutual context-aware trustworthy service evaluation model to assess service providers and consumers trustworthiness.

Authors in [83] applied only reputation and recommendation metrics that presented in [65] to propose their distributed trust model. Reputation metric in this study is redefined to be the opinion of other IoT nodes in the network and recommendation metric is redefined to be the opinions of social contacts. However, many other trust metrics are not considered.

Xiao et al. [64] proposed a centralized trust model for SIoT based on reputation by utilizing reputation server to store reputation information. Two parameters are used to model the trust in this study; QoS trust attribute called credit to get a service and social trust attribute called reputation to measure node’s trustworthiness. However, this study only considers the owner relationship and neglects other important social relationships.

### 8.2. QoS-Driven Trust Models

There are some trust models considering only QoS trust attributes. Chen et al. [84] proposed a trust model based on fuzzy logic where each node has a table for maintaining a data forwarding information acquired by overhearing activities of its neighbors. Their study considers only three QoS metrics including energy consumption, end-to-end packet forwarding ratio, and packet delivery ratio.

Wang et al. [85] and Lize et al. [86] proposed a centralized trust model and only considered QoS trust attributes in three network layers. The aim of trust model in the first layer, sensor layer, is to identify a subset of nodes for providing services based on their trust scores. Its aim in the second layer, core layer, is to acquire optimal routes in the network based on some attributes (e.g., historical trust, risk, service capability, ability of anti-attack, and recommended experience). While the aim in the third layer, application layer, is to select candidate trusted methods for data processing and storage based on some control attributes such as service efficiency, service history, and service risk.

Mendoza et al. [87] proposed a distributed trust model based on only direct information acquired by direct communication between IoT nodes. This work considers only service quality attribute. Their model assigns positive trust value to the node that fulfills a required service and negative trust value to the node that refuses to cooperate. The objective of their model is to mitigate OOA by utilizing a reward and punishment paradigm. Also, one QoS attribute is considered and no social attributes in their model. Later, Mendoza et al. [88]. extended their previous work presented in [87] by adding recommendations from their neighbors in the trust computation. The computation of the node’ trust involves two QoS trust attributes namely service quality and recommendation. They investigated the effectiveness of their model in the presence of only BMA.

Mahalle et al. [89] proposed a trust model for access control based on fuzzy logic considering the experience, knowledge, and recommendation QoS attributes. For the calculation of trust value, the model assigns linguistic values (e.g., good, average or bad) to the QoS attributes. To achieve access control in loT, the model maps the acquired fuzzy trust values to access permission.

Namal et al. [66] proposed an autonomic trust management framework based on a feedback control loop to assess the trust level considering only four QoS trust attributes namely, availability, response time, reliability, and capacity. The acquired trust values are disseminated in a centralized manner periodically.

Saied et al. [63] proposed a trust management system for IoT considering only service quality trust attribute with different context information. Their model assigns reputation score to cooperating nodes based on different context and different functions and uses a centralized manager to maintain all reputation reports sent by service providers after each transaction.

### 8.3. Social-Driven Trust Models

Guo et al. [44] proposed a 3-tier cloud-cloudlet-device trust model based on social relationships among owners of IoT devices. Three main social trust attributes are considered in this study namely, friendship, social contact, and community. Authors argue that users sharing same social relationships have similar views of the provided services by a trusted IoT device. Their model incorporates node’s observation and other IoT nodes’ weighted recommendations.

Azad et al. [70] proposed machine to machine reputation system to evaluate the trustworthiness of machines in IoT. Only reputation social trust metric is considered in this study. The participantsa ssign a trust value to the machine based on their experiences and interactions with the machine. Then, they send trust values’ cryptograms to the bulletin board. Utilizing secure multi-party computation methods, the reputation requester calculates the global reputation of machine by utilizing the reported cryptograms in the bulletin board.

Palaghias et al. [90] proposed an opportunistic sensing system called MobTrust to quantify and derive trust relationships among users through mobile phones by detecting real-world social interactions. In their system [90], trust assessment is based on various attributes that are taken out from users’ social interactions such as relative-orientation, frequency and duration of interactions. However, considering only these social attributes in the trust management model is not an effective solution because of the difference in assumptions on the association between the behaviors of the entities.

To conclude this section, we present classification to the existing IoT trust models based on trust design parameters and resistance to attack types in Table 4 and Table 5 respectively. We leave the reader with two summaries. Table 6 summarizes the simulation tools and metrics used to measure the performance of existing IoT trust models. Finally, Table 7 classifies IoT trust models based on the aggregation methods used in the trust computation phase.

## 9. Concluding Remarks

The IoT vision comes as a result of connecting heterogeneous nodes ranging from physical entities to smart devices. As stated in [42,43], there are 9 billion interconnected nodes and this number is expected to reach 20 billion by 2020. To benefit from the vision of IoT, data sources are the first step to be scrutinized. Usually, WSNs are utilized to gather and disseminate data. Data fusion techniques are then applied to discover desirable features or to enhance decision making. Finally, the fused data is consumed by smart applications in order to provide intelligent services.

Data sources’ misbehavior renders IoT smart applications unreliable. Therefore, during data collection and data fusion, we must select only trusted data sources and fusion centers. Since these sensors are the resource/information providers to fusion centers, a sensor could be captured by an adversary, which may lead to its non-cooperative behavior or misbehavior. In addition, a sensor can itself become untrustworthy. As such, IoT trust models are an essential requirement for information fusion and hence, for the success of IoT intelligent services. Designing an efficient architecture faces many issues such as heterogeneity, scalability, mobility, and constrained capabilities of various IoT entities.

Centralized IoT architectures [33,44,61,63,64,66,67,75], where a central trust manager computes and stores trust values, have common issues including single-point of failure and the difficulty of maintaining the global view of all involved IoT devices due to the dynamicity nature of IoT systems. Furthermore, traffic bottleneck is another issue when a large number of IoT sensors interact with the centralized trust manager consuming energy and disrupting communication bandwidth.

On the other hand, distributed trust models for IoT environments can be divided into three class, namely object-layer-based, fog-layer-based, or cloud-layer-based. Distributed architectures for modeling trust such as [68,69,70] implement the trust model at the things layer which has hardware constraints in terms of limited computing and energy resources. As such, these things will not be able to support basic functions like trust computation, trust propagation, trust updates, and trust storage [91]. To the best of our knowledge, the only distributed architecture that implements the trust model in the fog layer is [7]. This work allows smart objects to utilize the blockchain as storage to advertise their trust levels about other nodes. Scalability and mobility support of this architecture are inherited from the blockchain technology. Some functionalities, such as trust storage, of a trust model can also be implemented at the cloud layer. This implementation inherits similar disadvantages as the centralized IoT architectures.

## 10. Future Directions

### 10.1. Information Fusion and Trust

Conducting a reliable information fusion is an open field of research [36,92]. A data source might be dishonest, the fusion centre might be untrustworthy affecting the reliability of the fusion process itself, or the fusion center might act dishonestly affecting the fused data. As such, we need to consider trust in the data fusion notion. How to rely on data fusion processes would be a new area of research that unfortunately did not receive much attention from the research community. Furthermore, modeling trust should provide incentives to sensor nodes to participate as either data sources or fusion centers as long as they act in a trustworthy and honest manner.

Information fusion processes are being integrated into distributed systems as well as system-of-systems. Studies such as [93] apply a system-of-systems engineering process to obtain integrated architectures of information fusion systems. Distributed trust management systems have also been explored by many researchers [11,88,94]. A future direction is to apply a system-of-systems engineering process to obtain integrated architectures of trust-based information fusion systems.

### 10.2. Fog-Based Distributed Trust Models

IoT smart objects are deployed as low power, memory, and processing data sources to provide smart application domains such as smart homes and smart health care systems. To fully rely on these smart applications, objects need to be trustworthy and honest. Various trust models exist that can effectively cope with trust attacks but are not suitable for IoT as they incur high consumption of resources. One way to address this problem is by offloading the trust-related operations to a more resourceful entity such as a fog-based node. Generally, fog computing enables trust operations to be done directly at the network’s edge.

Hence, new IoT architectures for modeling trust should be proposed. These architectures must be efficient, scalable, and support mobility as well as taking into consideration the constrained capabilities of IoT devices. Furthermore, distributed architectures at the fog layer should be further investigated. Such architectures can avoid the bottleneck created at the cloud layer as well as avoiding the constrained capabilities at the things layer. Fundamental trust functionalities such as trust computation, trust storage, and advertisement can be done by the fog layer. In addition, these fog nodes can interact with each other to avoid visiting the cloud. For example, the fog-based methods for propagating trust levels received insufficient consideration [13,95]. So in order to address this research gap, fog-based distributed trust models need to be explored further.

### 10.3. Dynamics of Trust

When computing direct trust and reputation, the trust may decay with time. For example, if *x* trusts *y* at a given trust level based on experience five years ago, *x*’s trust in *y* today is likely to be lower unless they have continued to interact since then. Therefore, a decay function needs to be applied when obtaining direct trust levels or when giving recommendations. There are some issues that need to be sorted out before the decay function can be simulated and examined. First, how does the decay function apply to the trust levels. We need to explore the issue of quantity versus time. That is, by how much a trust level should be decayed and what is a reasonable time interval to decide applying decay function. Second, should there be a generic decay function mechanism and leave the implementation details to each individual node. Also, what is the trade offs in implementing a generic decay function that is used by all the nodes versus individual node’s decay implementations. Finally, how do the different implementation approaches of the decay function affect the overall performance of the trust model.

### 10.4. Using Trust Decay to Shape the Recommender Set

Since node reviews play a vital role in estimating the trust level, recommenders are a very important component in any trust model. Therefore, trust models should shape the set of recommenders and the objective is to have honest set of recommenders. Furthermore, having honest recommenders can give misleading reviews. Suppose that *z* is a recommender that *x* uses to collect reviews about *y*. At this point, let us assume that *z* is honest. If *z* is inactive and has not interacted with *y* for a long time, *z* trust level in *y* becomes stale. When *x* receives recommendations from *z*, these recommendations maybe as misleading as recommendations received from a dishonest node. Therefore, recommenders should be active as well as honest. This scenario emphasizes and illustrates the importance of further investigation to integrate decay functions with trust models.

## Figures and Tables

**Table 1:** Trust-related attack types with their descriptions.

Attack Type	Description
Self-promotion attack (SPA)	A dishonest node provides good recommendations for itself to promote its importance in order to be selected as a service provider. Then, it exploits its reputation to provide malicious service. An example of this attack occurs when a dishonest node positively fabricates fake feedback about itself or adjusts its own reputation during data dissemination.
Bad-mouthing attack (BMA)	A dishonest node can ruin the trust level of well-behaved nodes by giving bad recommendations about them. Consequently, their reputation is negatively affected and the chance of these well-behaved nodes to be selected for service is reduced.
Ballot-stuffing attack (BSA)	A dishonest node can boost the trust levels of other untrustworthy or dishonest nodes by giving good recommendations. As such, boosting their reputation.
Opportunistic service attack (OSA)	An untrustworthy node with a bad reputation may provide good service at a certain time to improve its reputation.
Collusion attack (CA)	This attack occurs when one or more nodes conspire together to defraud the trust level of one or more nodes.
On-off attack (OOA)	An untrustworthy node can randomly perform trustworthy service to hide its untrustworthy behavior.
Whitewashing attack (WWA)	An untrustworthy node can disappear and rejoin the application to wash away its bad reputation.
Discriminatory attack (DA)	An untrustworthy node can discriminate against specific nodes.

**Table 2:** Classification of trust-based information fusion approaches.

Ref.	Trust Integration			Target Node		Discounting of Reports	Fusion Operator/Method			
	Selection	Fusion Process		Source	Aggregator		Cumulative	Averaging	Consensus	Others
[57]	√			√			√			
[58]	√			√			√			
[46]	√			√		√	√			
[53]		√		√						√
[51]	√			√		√	√			
[49]		√		√						√
[52]		√		√						√
[60]		√		√	√	√				√
[59]	√			√			√	√		
[21]	√			√		√		√	√	
[45]	√			√		√			√	

**Table 3:** Summary of existing IoT architectures for modelling trust.

		Architecture Type		
		Centralized	Distributed	Hybrid
	Things	[63,64]	[68,69,70]	
IoT Layer	Fog	[36]	[7]	[44]
	Cloud	[33,61,65,75]		
Not Specified		[20]		

**Table 4:** Classification of IoT trust models based on trust design dimensions.

Ref.	Trust Components		Trust Attributes (Involvement)			Trust Discovery		Trust Advertisement			Trust Attributes (Quantity)	
	Direct	Rep.	Node (QoS)	Node (Social)	Network (QoS)	Dis.	Cen.	Time	On-Demand	Event	Single	Multiple
[68]	√	√	√	√		√				√		√
[39]	√	√	√	√		√				√		√
[69]	√	√	√	√		√				√	√	
[82]	√	√	√	√		√		√		√	√	
[84]	√	√	√		√	√		√			√	
[81]	√	√	√	√		√		√		√		√
[86]	√	√	√		√		√		√			√
[89]	√	√	√			√		√			√	
[87]	√		√			√				√	√	
[66]	√		√				√	√			√	
[79]	√	√	√	√		√	√			√		√
[63]		√	√				√			√	√	
[64]		√	√	√			√			√		√
[65]	√	√	√	√			√			√	√	
[70]		√		√		√				√	√	
[44]	√	√		√		√	√	√				√
[88]	√	√	√			√				√	√	
[76]	√	√	√	√		√				√	√	
[77]	√	√	√	√		√				√		√
[80]	√	√	√	√		√				√		√
[11]	√	√	√	√		√						√
[85]	√		√				√		√		√	
[90]	√			√		√				√		√

Ref. = Reference, Rep. = Reputation, Dis.= Distributed, Cen. = Centralized.

**Table 5:** Classification of existing IoT trust models based on resistance to attack types.

Ref.	Trust-Related Attack						
	SPA	BMA	BST	OSA	OOA	WWA	DA
[68]	√	√	√				
[39]	√	√	√			√	√
[69]	√	√	√	√			
[82]	√	√	√	√			
[84]	√						
[87]					√		
[79]	√	√	√	√			
[63]		√		√	√		
[64]				√			
[70]			√				
[44]	√	√	√	√			
[81]	√	√	√				
[88]		√					
[76]	√	√	√				
[80]	√	√	√	√		√	
[11]	√	√	√		√		

**Table 6:** Performance simulation tools and metrics used in the existing IoT trust models.

Ref.	Simulation Tool					Simulation Metrics			
	NS2	NS3	Cooja	Matlab	Others	Accuracy	Convergence	Resiliency	Others
[68]					√	√	√	√	
[39]		√				√	√	√	
[69]					√	√	√	√	√
[82]		√				√	√	√	
[84]		√						√	
[81]					√	√			√
[89]	√								√
[87]			√					√	
[66]				√		√			
[64]					√				√
[70]					√				√
[88]			√						√
[44]		√				√	√	√	
[76]					√	√	√	√	
[77]					√				√
[80]					√	√	√		
[11]					√	√		√	
[79]					√				√
[63]					√			√	

**Table 7:** Classification of existing trust models based on aggregation methods.

Aggregation Method					
	Bayesian	Fuzzy	Static	Dynamic	Utility
	Systems	Logic	Weighted Sum	Weighted Sum	Theory
**Ref.**	[69,82]	[65,84,89]	[39,44,66,68,76,77,79,80,81,84,86,87,88,89]	[63,69,82,90]	[65]

## Abbreviations

The following abbreviations are used in this manuscript:

IoT	Internet-of-Things
SIoT	Social Internet-of-Things
SOA	Service oriented architecture
WSN	Wireless sensor network
MANET	Mobile ad hoc network
